# Influence of Electropulsing Treatments on Mechanical Properties of UNS S32750 Duplex Stainless Steel

**DOI:** 10.3390/ma13071613

**Published:** 2020-04-01

**Authors:** Claudio Gennari, Luca Pezzato, Gianmarco Tarabotti, Andrea Zambon, Andrea Di Schino, Irene Calliari

**Affiliations:** 1 Department of Industrial Engineering, University of Padua, Via Marzolo 9, 35131 Padua, Italy; luca.pezzato@unipd.it (L.P.); gianmarco.tarabotti@studenti.unipd.it (G.T.); irene.calliari@unipd.it (I.C.); 2 Department of Management and Engineering, University of Padua, Stradella S. Nicola 3, 36100 Vicenza, Italy; a.zambon@unipd.it; 3 Department of Engineering, University of Perugia, Via G. Duranti 93, 06125 Perugia, Italy; andrea.dischino@unipg.it

**Keywords:** electroplastic effect, pulsed current, duplex stainless steel, electropulsing treatment, tensile test

## Abstract

Prestrained at 5% and 15% duplex stainless steel UNS S32750 specimens have been subjected to electropulsing treatments with current density of 100 A/mm^2^ and 200 A/mm^2^ and 100 and 500 pulses for each current density value. Corrosion tests, X-ray diffraction, microhardness and residual stresses were collected before and after the electropulsing treatments. Tensile tests were performed after the electropulsing treatments in order to compare the mechanical response to reference tensile tests performed before pulsing treatments. Increase in fracture strain was observed after pulsing treatment in comparison to the reference tensile tests. A decrease in microhardness was also observed after electropulsing treatments for both degrees of prestrain. Electropulsing treatment almost eliminates the work-hardened state in the 5% prestrained specimens while partially recovered the 15% prestrained material increasing both uniform and fracture strain. Bulk temperature of the samples remained the same for all treatments duration. The effect are to be addressed to a combined effect of increase in atomic flux due to the electrical current and local joule heating in correspondence of crystal defects. Electropulsing treatment applied to metallic alloys is a promising technique to reduce the work hardening state without the need of annealing treatments in a dedicated furnace.

## 1. Introduction

Duplex stainless steels (DSSs) are a peculiar category of stainless steels characterized by their biphasic microstructure consisting of almost equal volume fraction of austenite and ferrite. They are employed in different applications, such as oil and gas, paper and pulp industry, the wine industry, etc. [1,2,3]. Their mechanical and corrosion properties are higher compared to austenitic stainless steels, on the other hand they suffer from secondary phase precipitation which causes embrittlement, poor corrosion properties and limits their working temperature to 350 °C [4,5,6,7,8,9,10,11,12].

A balanced microstructure consisting of equal amounts of austenite and ferrite is necessary in order to obtain the best combination of mechanical and corrosion properties. This can be achieved with a suitable composition and a solution treatment performed at a temperature that depends on the steel composition (however higher than 1050 °C). The solution treatment is also mandatory in order to dissolve any possible detrimental secondary phases that might have precipitated during previous forming processes and heat cycles [13]. Secondary phases in DSSs precipitate in a temperature range between 600 °C and 1000 °C. Spinodal decomposition of ferrite into high chromium content ferrite and lower chromium content ferrite could take place at lower temperature (approximately 475 °C) if the soaking time is high enough because of the slow decomposition kinetic. In high alloyed DSSs, such as UNS S32750 and UNS S32760, secondary phase precipitation takes place in a matter of minutes; it is therefore necessary to limit their working temperature below 475 °C and perform a solution treatment after high temperature forming processes (hot rolling, forging etc.). Most common secondary phases found in DSSs are χ phase, σ phase and chromium nitrides (i.e., CrN and Cr_2_N) and the less common π-phase, Laves phase, R-phase and carbides. The first phase to precipitate is χ phase due to its lower coherency strain with the cubic lattice of ferrite even though σ phase is thermodynamically more stable [5].

The effect of electrical current during plastic deformation of materials was discovered by Machlin in 1959 [14]. The discovery of such phenomenon led to the development of new approaches to material forming known as electrically assisted manufacturing (EAM) in which electrical current increases the formability of various metallic alloy exploiting the electroplastic effect (EPE). The electroplastic effect has shown to improve the formability on a wide variety of metallic materials such as aluminum [15,16,17], titanium [18,19,20], magnesium [21,22,23], stainless steels [24,25] and on a variety of forming processes as well. Some of the authors observed a relationship of the onset of EPE on FCC materials with respect the stacking fault energy (SFE), which drives dislocation’s motion within the material [26]. Conversely to EAM, during electropulsing treatments (EPTs), electrical current is applied prior or after deformation. EPTs have been shown to influence mechanical and microstructure properties of different metallic alloys [27]. Ben et al. were able to rapidly harden AISI 4340 steel due to a combined effect of dislocations, solid solution strengthening and nano twinned martensite thanks to EPT [28]. Guan and Tang observed an evolution of the texture in cold rolled AZ31 magnesium alloy and a grain refinement after EPT [29]. Xiang and Zhang dramatically reduced residual stresses on the surface and inside of as quenched samples of a pipeline steel after electropulsing treatment [30]. Luu et al. were able to perform rapid annealing on AISI 316L with a single current pulse in between two forming processes increasing its formability [31]. Sànchez et al. were able to reduce the force and the backspring during bottom bending process on aluminum and stainless steel [32]. Many theories have been developed in order to identify the mechanisms that electrical current produces on the microstructure, but a unanimous consensus has not yet been reached.

Some of the mechanisms induced by the electrical current are: electron wind force (transfer of momentum of conducting electron to dislocations increasing their mobility) [33], magnetoplastic effect or magnetoplasticity (depinning of dislocations from weak obstacles thanks to the induced magnetic field) [34], electron stagnation theory (localized change in resistivity causes an increase of electron to atom ratio, weakening the metallic bond and easing its breaking and restoration) [15], electromigration (increase of ions diffusivity thanks to the electrical current ) [35], reduced Gibbs free energy during phase transformation [36,37] and localized microscale Joule heating [38].

Investigations of the effect of both EAM and EPT on DSSs are still quite limited except for the work carried out by some of the authors [24] and a scientific report on duplex steel by Rahnama et al. [39]. The aim of the present work is to examine the effect on the mechanical properties of electropulsing treatments on a material that possesses two phases with different crystal structure, composition, electrical resistivity, work hardening rate etc. The opportunity to perform EPTs between forming processes and avoid heating the material in the secondary phases’ temperature stability regime could aid to improve processing of these steel grades.

## 2. Materials and Methods

The material was supplied by the Italian division of Outokumpu S.p.A. in form of 2 mm thick warm-rolled metal sheet. The composition of the investigated steel is reported in Table 1.

Specimens for tensile tests were obtained along transverse direction with geometry according to ASTM E8 standard except for the gauge length which was reduced to 45 mm due to the small dimension of the metal sheet. Secondary phase identification, phase balance quantification and profile fitting were conducted through X-ray diffraction pattern acquisition on a Bruker D8 X-ray diffractometer (Bruker Corporation, Billerica, MA, USA) equipped with Cu X-ray tube without monochromator on the detector side. Scan steps of 0.02° and counting time of 5 s were used for X-ray diffraction measurements. The evaluation of the volume fraction of the constituting phases was performed through Rietveld analysis on the as-received material by means of MAUD^©^ Software (Luca Lutterotti, Trento, Italy).

Tensile tests were conducted at a strain rate of 10^−2^ s^−1^ (TRIP effect was observed at lower strain rate) on an MTS 322 tensile test machine (MTS System Corporation, Eden Prairie, MN, USA) capable of a maximum force of 50 kN modified to perform electroplasticity tests. Force was measured through the load cell of the MTS while displacement through the crosshead movement.

**Figure 1:**
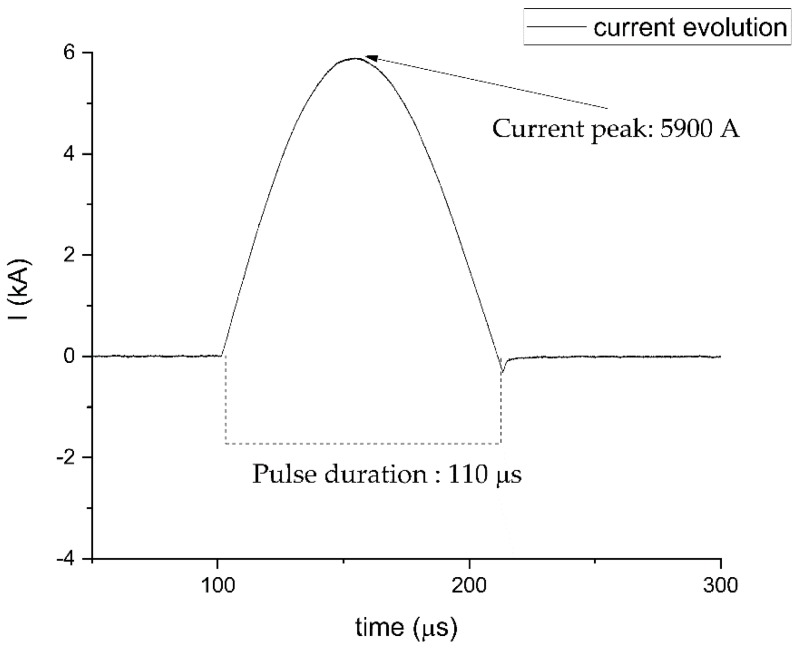
Waveform and duration of the typical electrical current pulse delivered by the power supply.

A self-designed power supply capable of delivering electrical pulses of duration of 110 µs at maximum frequency of 50 Hz and up to 6 kA were used for the electropulsing treatments. The waveform of the electrical current is displayed in Figure 1. The temperature of the specimens was collected through a FLIR A40 infrared camera. The side of the specimen facing the infrared camera was painted with a black opaque lacquer in order to obtain a uniform emissivity distribution.

Microhardness measurements were performed on a Leitz Miniload 2 (Leica Microsystem S.r.l., Milan, Italy) microhardness tester with 500 g load for the bulk hardness and 25 g load for the individual phase.

Residual stress measurements were performed by means of the “sin^2^ψ method”. A dedicated X-ray diffractometer SpiderX (G.N.R. S.r.l., Agrate Conturbia, Novara, Italy), equipped with Cr tube radiation, was used to acquire the necessary X-ray pattern for both the ferritic and the austenitic phases in the rolling as well as the transverse direction. To this end {222} reflection for ferritic phase and {321} reflection for austenitic phase have been chosen. Thirteen psi angles varying between −45° and +45° with a counting time of 300 s were utilized. To compute the residual stresses, elastic constants values for ferrite and austenite were utilized according to Johansson et al. paper [40].

Potentiodynamic polarization tests were performed on an AMEL 2549 potentiostat (Amel Electrochemistry, Milan, Italy) at a scanning rate of 0.5 mV/s in a 0.01 M NaCl solution with a saturated calomel electrode as reference electrode and a platinum electrode as counter electrode, according to standard ASTM G3-14. The potentiodynamic polarization were performed in a potential range between −0.8 V and 1.2 V, after stabilization of the open circuit voltage (OCP) for 30 min. Each test was repeated three times in order to assure reproducibility.

Microstructural investigations were carried out through optical microscopy (Leica DMRE, Leica Microsystems S.r.l., Milan, Italy) and scanning electron microscopy (Leica Cambridge Stereoscan LEO 440, Leica Microsystems S.r.l., Milan, Italy) operating in back scattered electron (BSE) at 29 kV. Ordinary metallographic preparation was carried out. To reveal the microstructure, electrolytic etching with NaOH at 3 V for 5 s was used.

The specimens were divided into two main categories. Room temperature tensile tests were performed to obtain the reference mechanical properties, focusing on the uniform strain. Specimens of the first category were strained to 5% while the ones of category number two were strained to 15%. After straining, residual stresses along the two directions and on the two phases, X-ray diffraction measurements, corrosion tests, microhardness measurements and microstructural analysis were conducted. Three specimens per category were then strained until fracture in order to have a reference for the specimens which were to be strained after electropulsing treatments.

Each category was then subdivided into groups depending on the electrical parameters:

- Group 1: 100 A/mm^2^ 100 pulses
- Group 2: 100 A/mm^2^ 500 pulses
- Group 3: 200 A/mm^2^ 100 pulses
- Group 4: 200 A/mm^2^ 500 pulses

As for the as supplied reference material, after each electropulsing treatment, residual stresses along the two directions and on the two phases, X-ray diffraction measurements, microhardness measurements and microstructural analysis were conducted.

**Figure 2:**
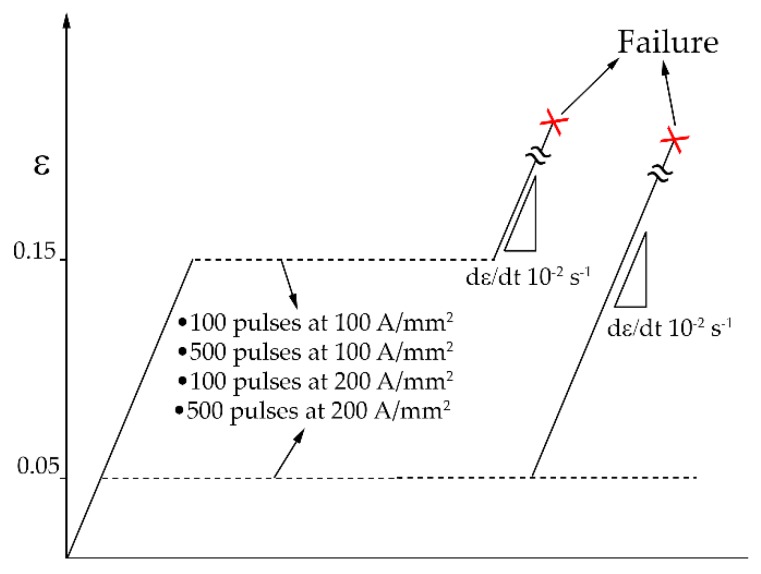
Schematic of the experimental procedure.

Subsequently specimens of the two categories were strained until fracture and compared to the reference test of each category. A schematic of the experimental procedure is showed in Figure 2.

## 3. Results

### 3.1. As-Received Material

The as-received material showed a very well-balanced microstructure free of secondary phases, which was confirmed both by X-ray diffraction patterns examination (only peaks of δ-ferrite and γ-austenite were visible) and to SEM-BSE investigations.

**Figure 3:**
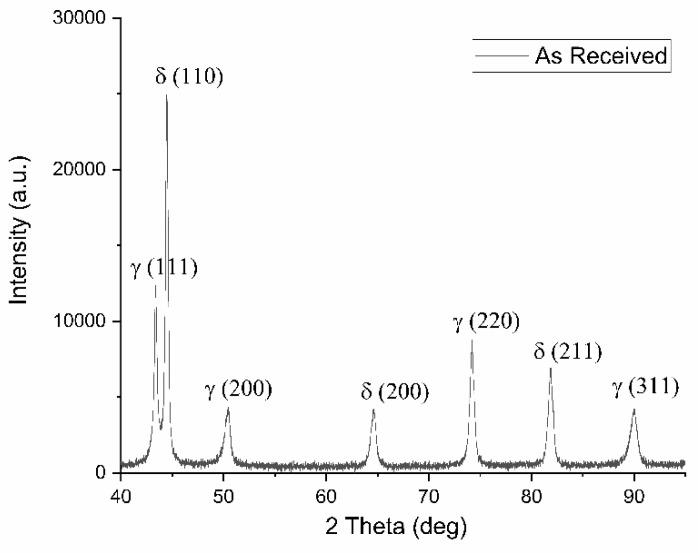
X-ray diffraction pattern of the as received material along rolling direction.

Phase balance was evaluated through Rietveld analysis performed on the X-ray diffraction pattern of Figure 3 and resulted in almost equal volume fraction of austenite and ferrite (0.48 ± 0.04 and 0.52 ± 0.05 respectively).

**Figure 4:**
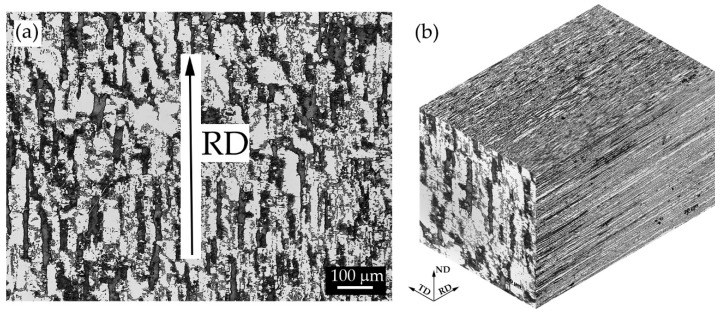
Microstructure of the as received materials: (**a**) along the rolling direction (RD) and (**b**) along the main three directions (**b**). Etching solution NaOH at 3 V and 5 s.

The as received material is characterized by a microstructure consisting of fragmented austenite islands (bright grains) dispersed in a ferritic matrix (dark grains) as can be seen in Figure 4a. Rolling direction is highlighted by the black arrow. Austenite morphology was quite fragmented due to the last pass of the rolling process which was conducted at a lower temperature. Microstructure along the other dimensions (transversal and normal) is shown in Figure 4b. The interphase space (space between austenite and ferrite grain centres) was smaller along the normal direction in comparison to that along the rolling direction.

### 3.2. Reference Tensile Tests

**Figure 5:**
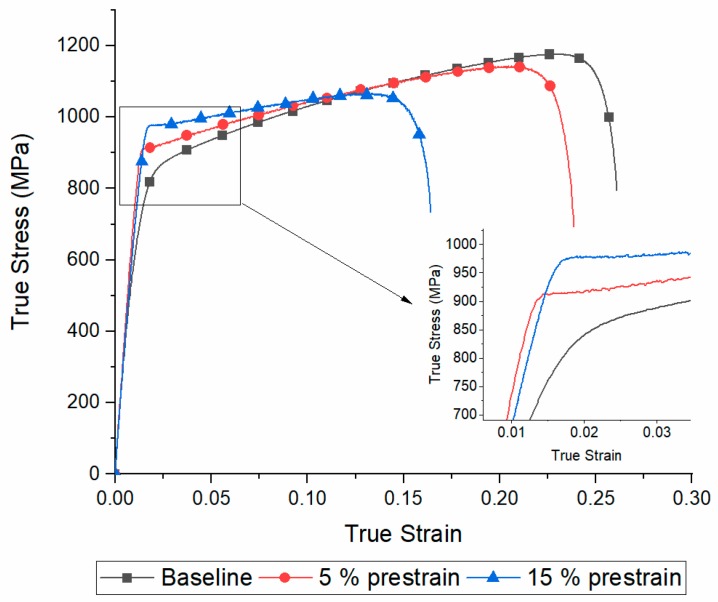
True stress-strain curves of the as received material and the two categories of specimens prestrained at 5% and 15%.

Tensile tests on the as received material and on the specimens prestrained at 5% and 15% were conducted in order to have a reference to compare the tensile behaviour of the electropulsed specimens after EPTs (Figure 5).

As the prestrain amount increased lower values of fracture and uniform strain were observed, while yield stress (YS) and ultimate tensile strength (UTS) increased.

**Figure 6:**
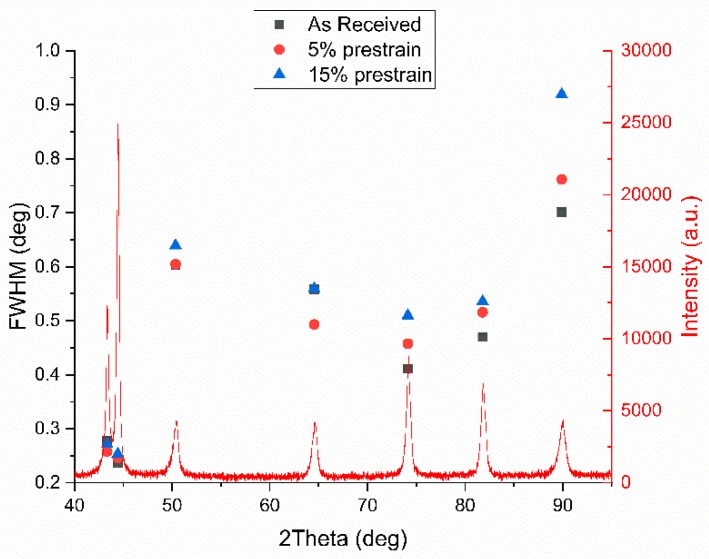
X-ray diffraction pattern together with full width half maximum values of the set of three samples.

X-ray diffraction patterns of the three samples were collected before straining until fracture and FWHM are reported in Figure 6 (▪, •, ▲ symbols).

**Figure 7:**
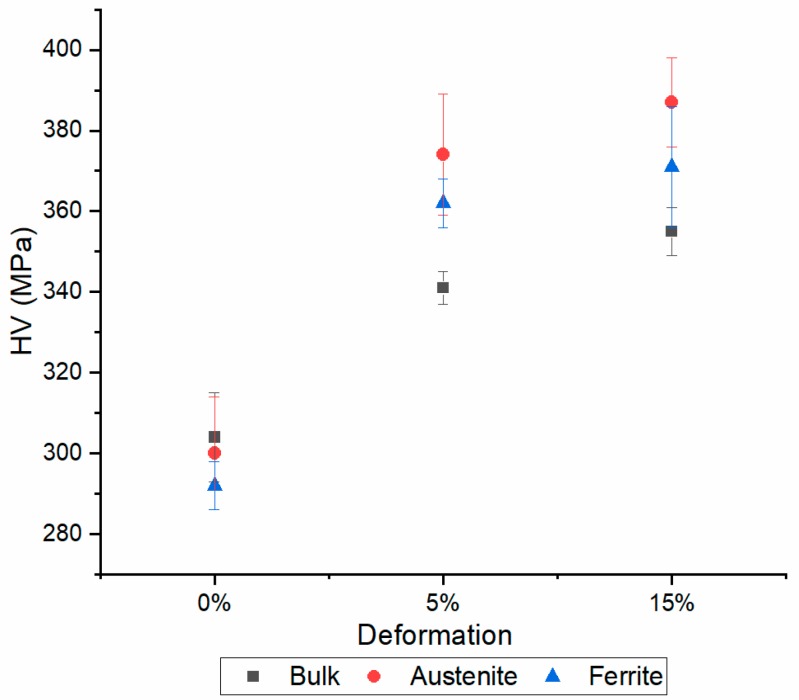
Microhardness evolution as a function of prestrain of the bulk material and of each phase.

Peaks broadening is affected by dislocation density, microstrain, stacking faults, crystallite size etc. [41,42,43,44,45,46,47]. It is therefore very difficult to separate the contribution of each phenomenon, so raw FWHM values are considered. As expected, as the prestrain increased FWHM increased too, because of the higher dislocation density, the generation of stacking fault in the austenitic phase, the evolution of the crystallite size due to dislocation network, etc. The higher the prestrain the higher the microhardness as well (Figure 7).

Microhardness of the bulk material was lower compared to that of the single phases because it was measured with a higher load. The smaller the load the more the measure is affected by the metallographic preparation which produces a shallow work hardened layer at the surface not to mention the indentation size effect. As the prestrain increased, the microhardness of the austenitic phase increased due to its higher strain hardening rate because of the lower YS in comparison to that of ferrite [48,49].

**Figure 8:**
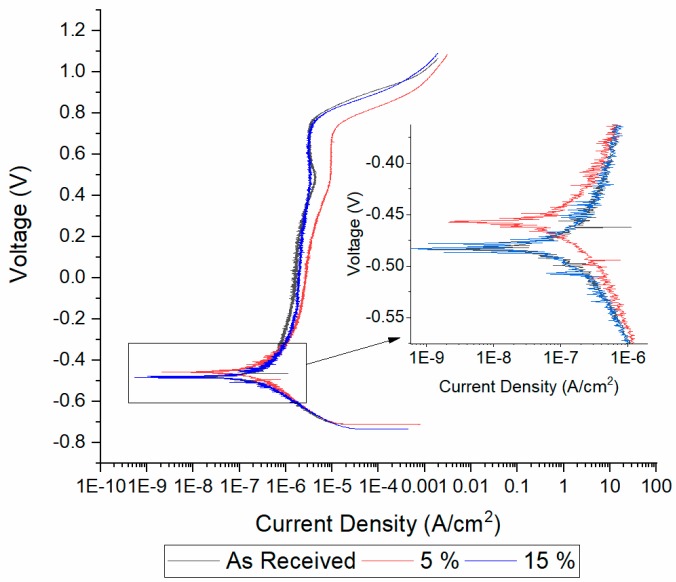
Potentiodynamic polarization curves of tested materials.

Corrosion properties were not substantially affected by the degree of prestrain as can be seen in Figure 8, where the potentiodynamic polarization plots of the as received and prestrained at 5% and 15% samples are shown. The only observed effect was the raise in corrosion potential of the 5% prestrained specimen in comparison to the others. No effect on the corrosion current were observed.

**Figure 9:**
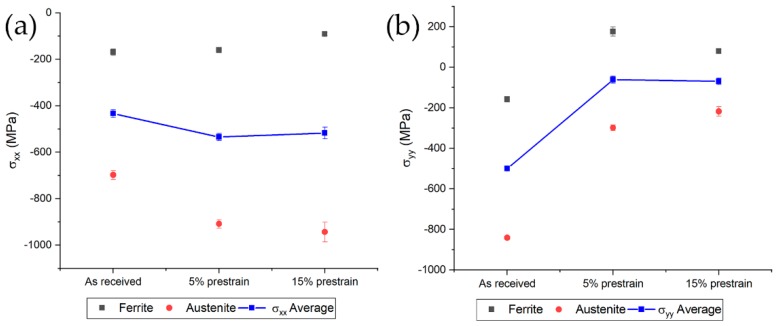
Residual stresses evolution on the single phases and the average along the rolling direction (**a**) and the transversal direction (**b**).

Compressive stresses in each phase along the two directions were measured, conversely to what Johansson et al. found [40]. A decrease in the compressive stress values for both phases were determined since the specimens were strained along the transversal direction (Figure 9b). The average stress was computed with the rule of mixture according to [40]. Compressive stresses along in single phases along rolling direction increased in austenite and decreased in ferrite. Average residual stress remained almost constant as expected since a tensile test produces the superposition of a uniaxial stress condition on the previously existing stresses distribution in the specimen.

### 3.3. Tensile Tests

Electropulsing treatments were conducted with current density of 100 A/mm^2^ and 200 A/mm^2^ at 1 Hz for 100 s and 500 s. No increase in specimen temperature was observed during the electropulsing treatments due to the small duration and low frequency of the current pulses.

**Figure 10:**
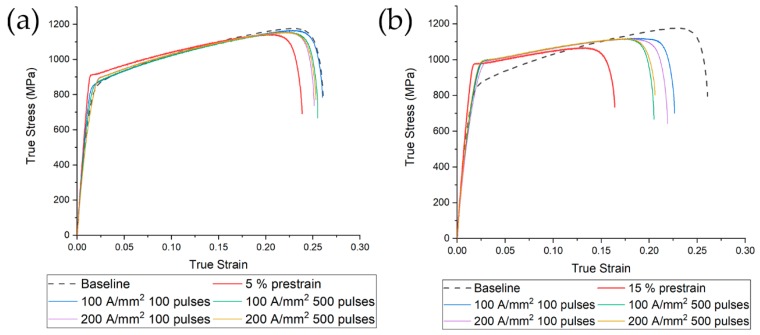
Tensile tests of electropulsed specimens after prestrain of (**a**) 5% and (**b**) 15%. The dashed line represents the room temperature test, while red line is the reference of each categories (5% tensile test (**a**) and 15% tensile test (**b**)).

After electropulsing treatments, tensile tests have been conducted and compared with the reference of each category (namely 5% tensile test curve of category one and 15% tensile test curve of category two, together with the flow stress curve of the tensile test performed on the undeformed material, Figure 10). Overall improvement in mechanical properties has been observed for both categories. In the 5% prestrained specimens, the best combination of current density and number of pulses was the lowest (100 A/mm^2^ and 100 pulses). Nevertheless, all tested conditions affected the tensile response in a positive manner. In the case of specimens prestrained at 15% a recovery of approximately 8% in fracture strain was observed for all tested conditions. The best combination of number of pulses and current density was the same observed as in the 5% prestrain case (100 A/mm^2^ and 100 pulses). Comparing the effect of the different combinations of current-number of pulses it appears that mechanical properties are more affected by the number of pulses rather than the current density. Fixing the current density, as the number of pulses increased, small improvements in fracture strain were observed. The two current densities tested affected to a lesser extent the recovery of fracture strain in comparison to the effect of the number of pulses.

### 3.4. Microhardness

**Figure 11:**
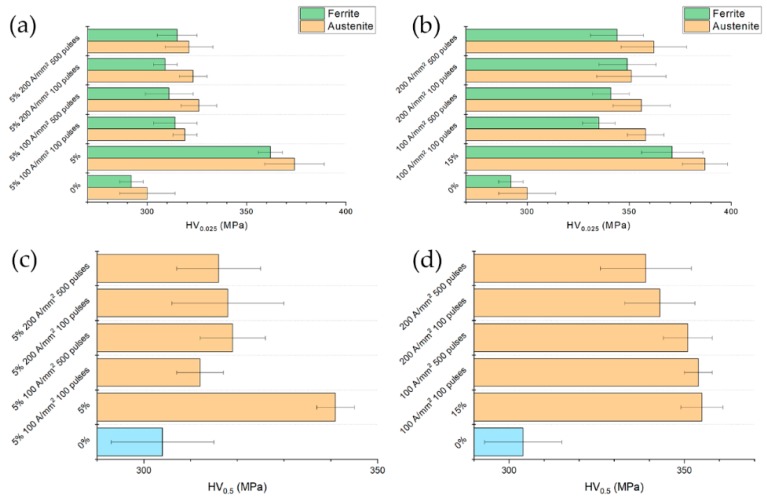
Single phase hardness and bulk hardness of specimens strained and electropulsed at (**a**,**c**) 5% and (**b**,**d**) 15% compared to the baseline (0%) and the related reference tests.

Bulk hardness of the electropulsed specimens of 5% prestrained material was slightly higher than that of the undeformed material (0%) while it was remarkably lower compared to the reference (Figure 11). Those results were expected on the basis of the true stress-strain curves of Figure 10a. Conversely, despite the substantial recovery of the specimens prestrained to 15% in comparison to the reference tensile tests, no appreciable bulk hardness reduction was observed. Electropulsing treatments conducted at higher current density showed a lower bulk hardness in contrast to what could be expected on the basis of flow stress curve of Figure 10b. Microhardness of the single phases showed the same trend as the bulk hardness. Austenite is the harder phase because of its higher work-hardening rate compared to ferrite. The reduction of hardness was almost the same for both phases.

### 3.5. Ultimate Tensile Strength and Yield Stress

**Figure 12:**
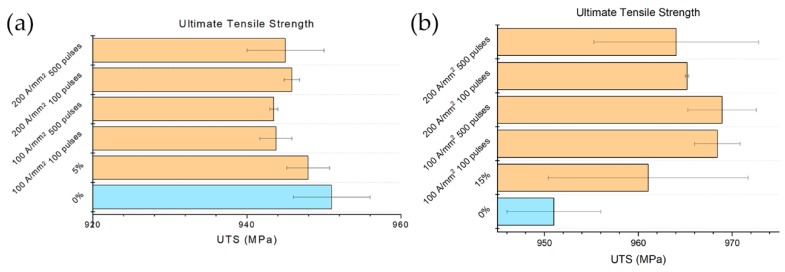
Evolution of UTS with respect the different electropulsing treatments and references (**a**) 5% prestrained specimens and (**b**) 15% prestrained specimens.

Ultimate tensile strength was affected to a much lesser extent by both prestrain and by the electropulsing treatment. As can be seen from Figure 12a, 5% prestrain had no effect on the UTS while the electropulsing treatments caused a slight decrease of UTS in comparison to that of the prestrained material. As opposed to what expected, the electropulsing treatments increased UTS in the case of the sample strained at 15% (Figure 12b). This is because the uniform strain of the reference specimen (ε_u_ = 0.184) was very close to the value of the prestrain applied to the specimens of category two (ε = 0.15). Therefore, electropulsing treatments were able to recover a lot in terms of fracture strain, allowing the material to accept a higher amount of deformation, hence increasing its ability to work-harden which consequently led to higher UTS.

**Figure 13:**
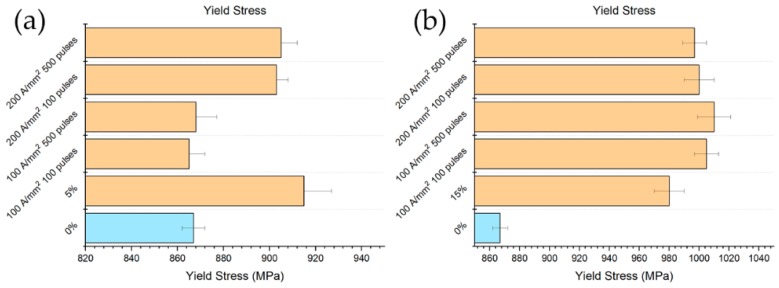
Evolution of YS with respect the different electropulsing treatments and references (**a**) 5% prestrained specimens and (**b**) 15% prestrained specimens.

The yield stress, in the case of 5% prestrain, was higher in comparison both to that of the reference and the electropulsed specimens, as expected. A substantial reduction was observed for the specimens electropulsed with lower current density, while a very low or even no reduction was measured with higher current density (Figure 13a). Unexpectedly yield stress increased in the case of the samples prestrained at 15% and electropulsed, even in comparison to the reference value (Figure 13b).

### 3.6. Uniform and Fracture Strain

**Figure 14:**
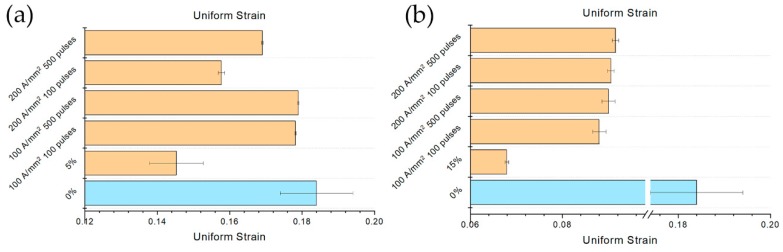
Evolution of uniform strain with respect the different electropulsing treatments and reference (**a**) 5% prestrained specimens and (**b**) 15% prestrained specimens.

**Figure 15:**
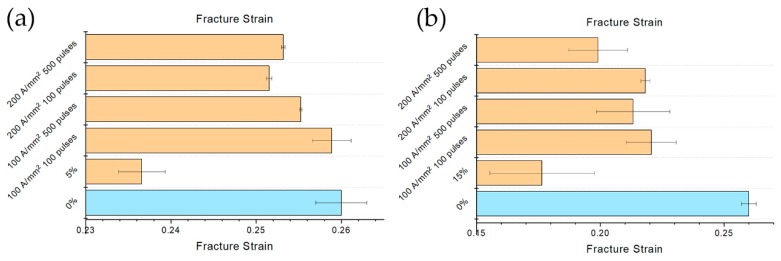
Evolution of fracture strain with respect the different electropulsing treatments and reference (**a**) 5% prestrained specimens and (**b**) 15% prestrained specimens.

The effect of the electropulsing treatments are much more evident considering the uniform and fracture strain rather than UTS and YS (Figure 14 and Figure 15). In the case of the specimens prestrained at 5%, uniform strain was almost the same of the reference specimen for the electropulsing treatments conducted at the lower current density. Nevertheless, even at 200 A/mm^2^ a substantial increase in uniform strain was observed in comparison to the untreated material. Much more evident was the effect of the electropulsing treatments on the specimens prestrained at 15%. The relative recovery in uniform strain was higher in this case compared to the previous one; a slight constant increase of the uniform strain increasing the current density and the number of pulses was observed (Figure 14b).

In the case of the specimen prestrained at 5% fracture strain did not show any trend (Figure 15a). As said before, the treatment at lower current density and lower number of pulses showed a fracture strain comparable to that of the reference specimen. The fracture strain of the specimens treated at 200 A/mm^2^, even if it was higher compared to the prestrained material, was slightly inferior to that of the 100 A/mm^2^ treatment, regardless of the number of pulses. It is clear from Figure 15b that, in the case of 15% prestrained specimens, the number of pulses affected in more extent the fracture strain rather than the current density. EPTs conducted with lower number of pulses showed the same increase in fracture strain. The most severe electropulsing treatment (200 A/mm^2^ 500 pulses) increased fracture strain in comparison to the prestrained material but was the worst in terms of absolute increase in fracture strain. It is worth noting the generally high scattering of the data regarding the test conducted on the specimens prestrained at 15%.

### 3.7. Residual Stresses

**Figure 16:**
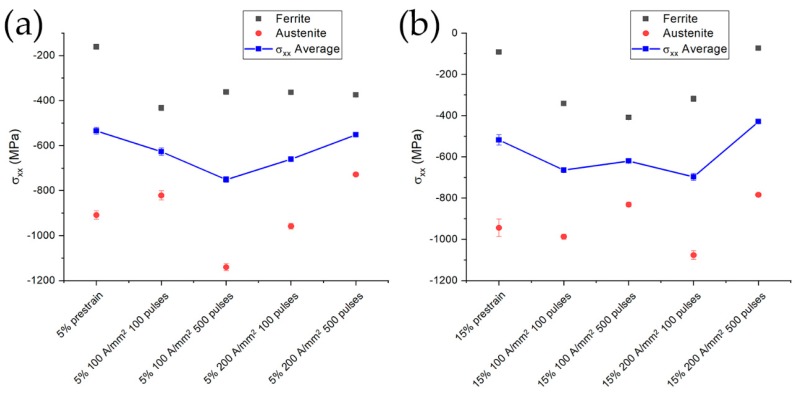
Evolution of transverse residual stresses in austenite (red circles), ferrite (black squares) and their average (blue thick line) for specimens prestrained and electropulsed at (**a**) 5% and (**b**) 15%.

The average transverse residual stresses for both degrees of prestrain showed almost the same values (Figure 16). Since tensile test introduces uniaxial stress configuration in the specimen, no variation in the average residual internal stresses was expected. On the other hand, electropulsing treatments have shown to induce changes in the grain orientation within the materials not to mention the change in morphology of secondary low conductivity phases (i.e., cementite particles in perlite) hence, a change in the residual stresses of the single phase was expected [30,50,51,52,53]. An increase in compression stress amounts in ferrite after the first electropulsing treatment was determined and this did not change even after the electropulsing treatment at higher current density and higher number of pulses (Figure 16a). In the case of the specimens prestrained at 15%, again an increase of the compressive state in ferrite (approximately from −50 MPa to −400 MPa) was determined for the lower current density treated samples. The higher current density treatment produced a decrease of the compressive stresses in ferrite from −400 MPa to almost the same value as the prestrained material (Figure 16b). The compressive state in austenite was much severe in comparison to that in ferrite (Figure 16a) but no significant trend have been observed. Same considerations can be made in the case of the material prestrained at 15% (Figure 16b). On the other hand, the average residual stresses in both cases remained almost constant.

**Figure 17:**
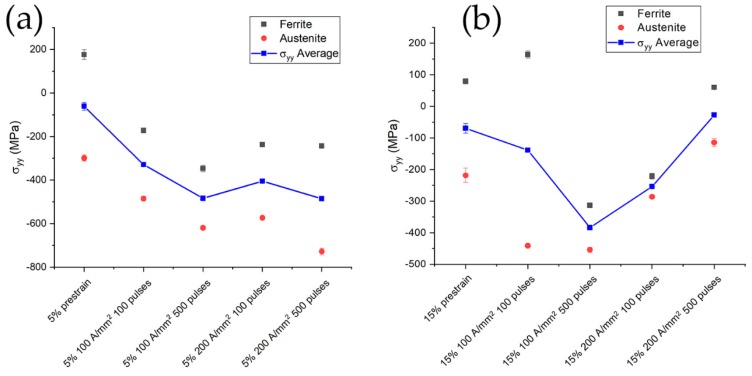
Evolution of longitudinal residual stresses in austenite (red circles), ferrite (black squares) and their average (blue thick line) for specimens prestrained and electropulsed at (**a**) 5% and (**b**) 15%.

Different evolution of the average residual stresses along the longitudinal direction can be seen in Figure 17. A gradual increase in the compression state in the case of the specimen prestrained at 5% was evident (Figure 17a) with a slight decrease for the specimens treated with 100 pulses at 200 A/mm^2^. The same trend was observed for the material prestrained at 15% under electropulsing treatment at 100 A/mm^2^, while substantial decrease in the compressive stress state for the treatments conducted at 200 A/mm^2^ were induced. Electropulsing treatments consisting of 500 pulses at 200 A/mm^2^ presented stress values and distributions comparable to that of the prestrained material. Residual stresses of the single phase followed the same trend of the average residual stress both for the specimens prestrained at 5% and 15% as can be seen in Figure 17. The only exception was the material prestrained at 15% treated with 100 pulses at 100 A/mm^2^ in which ferrite abruptly changed from a tensile stress value of approximately 150 MPa to a compressive value of 300 MPa when increasing the number of pulses.

### 3.8. X-ray Diffraction

X-ray diffraction measurements were conducted in order to evaluate the evolution of the peak’s broadening as a function of the specimen conditions.

**Figure 18:**
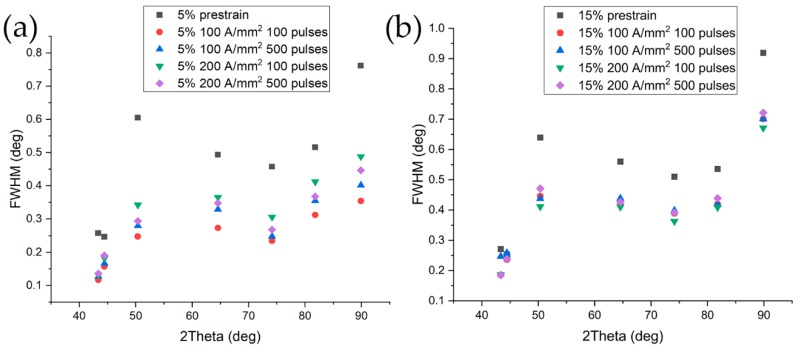
FWHM of the electropulsed specimens prestrained at (**a**) 5% and (**b**) 15%.

**Figure 19:**
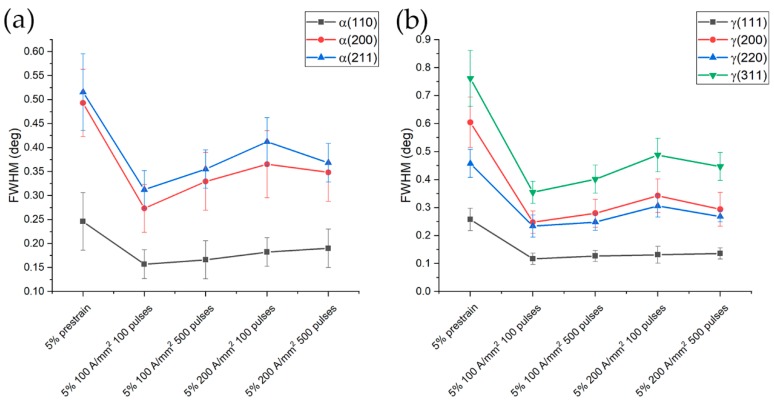
FWHM evolution of ferrite (**a**) and austenite (**b**) for the specimens prestrained at 5% and electropulsed.

**Figure 20:**
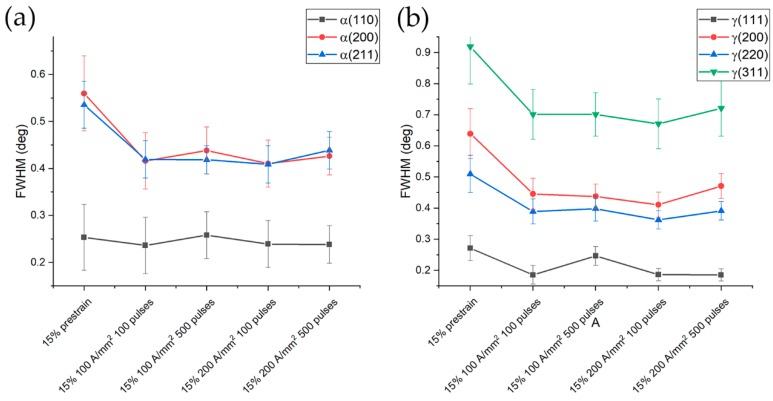
FWHM evolution of ferrite (**a**) and austenite (**b**) for the specimens prestrained at 15% and electropulsed.

An overview of the evolution of the FWHM of the peaks related to the different treatment conditions is reported in Figure 18. Being the FWHM influenced by dislocation density, microstrain, stacking fault density, etc., it is obvious that the higher value of the FWHM is found in the specimens prestrained at 5% and 15% (black squares in Figure 18). In order to enhance the differences in FWHM of each phase, a separate diagram was built (Figure 19 and Figure 20).

As expected from the tensile test curves, the most significant decrease in FWHM of the 5% prestrained samples, was observed in the case of the treatments that showed the highest recovery in fracture strain (100 pulses at 100 A/mm^2^ in Figure 19). A slight increase in FWHM was observed with the other electropulsed treatment, with a subsequent reduction for the electropulsing treatment at higher current density and number of pulses. It is interesting to note the higher reduction in terms of absolute values for the FWHM relative to the austenitic phase, due to its higher dislocation density in the as strained condition because of the low yield point and higher work hardening rate compared to ferrite.

The reduction of FWHM in the case of the 15% prestrained samples was lower in comparison to that of the samples prestrained at 5%. Same trend both for the ferritic and austenitic phase was observed (Figure 20). FWHM remained almost constant regardless the current density and number of pulses, except for a slight increase after 500 pulses at 100 A/mm^2^ (more evident in Figure 20a).

## 4. Discussion

It is mandatory to know how duplex stainless steels behave during tensile test to understand the microscopic changes in dislocation network, strain and stress distribution that can affect current flow.

Due to the lower yield stress, austenite is the phase in which most of the strain is localized at the beginning of deformation. It absorbs much of the early stage deformation because it possesses 12 low-shear strength preferred slip systems and allows easy dislocation mobility in almost all crystallographic directions while ferrite is characterized by relatively higher-shear strength slip systems, which typically possess higher critically resolved shear stresses for their activation [54]. Even though ferrite has more slip system, its Peierls stress is higher compared to the yield stress of austenite, justifying the fact that austenite is more ductile than ferrite. Ferrite starts to accommodate plastic deformation after austenite has work-hardened enough to increase its yield stress above that necessary to overcome the Peierls barriers in ferrite. After that, ferrite starts to accommodate higher plastic strain than austenite, while some austenitic grains experience a more pronounced increase in the hardening effect depending on the orientation relationship between austenite and ferrite (Kurdjumov–Sachs orientation is the most favourable for the transfer of strain field between austenite and ferrite [10,55,56,57]). Direct dislocation transfer between austenite and ferrite is obviously impossible due to the phase boundary, but dislocations on the austenitic phase that keep piling up on the phase boundary could generate dislocation sources in the adjacent ferritic phase. It is therefore clear, that phase boundaries play an important role in governing the deformation mechanism of duplex stainless steels. 

They are also inhomogeneities, filled with dislocations and stacking faults which increase the local resistivity. When electrical current passes through the material, macro and microscopic effect occur. Bulk joule heating is a macroscopic effect, while at microscopic level electrons from electrical current interacts with the inhomogeneities in the microstructure. As modelled by Zhao et al. [38], electrical current does not flow homogeneously throughout the microstructure: if secondary phases are not present, it is influenced by the grain boundary network. The grain boundary network acts on the electrical current, forcing it to flow across an “easy path”, such as the triple junctions. As a consequence, the current flow is affected by the angle between the macro current flow and the grain boundaries [38]. This uneven distribution of electrical current could cause local increase in current density which can enhance plastic deformation thanks to localized Joule heating and to the effect of a stronger electron wind force on dislocations.

In this specific case, on top of grain boundary network, another phase with different crystal structure, dislocation density and composition is present. It is therefore legitimate to hypothesize an uneven distribution of the current within the material. In correspondence of regions with different electrical resistivity (grain and phase boundary, dislocation tangles, dislocation sub cell walls etc.) there could be stagnation of electrons as proposed by Ruszkiewicz et al. [15]. Electron stagnation could cause local increase in electron to atom ratio, leading to lowering the bonding energy between the ions of the crystal structure therefore easing dislocation motion in the case of current applied during deformation [58]. In this case the effect of electric current facilitates the recovery of the work-hardened state thanks to localized Joule heating effect, the reduction in bonding energy and the increase in atomic flux due to electromigration [35,59,60]. Electrical current will be unevenly distributed at multiscale levels: at microscopic level dislocation tangles, sub cell walls, grain boundaries etc., affect the current distribution while at an intermediate scale the different resistivity, composition and work hardening state of the two phases could lead to partial redistribution of the current within one phase or the other. All the above mentioned phenomena are concurrent and caused the recovery of the work-hardened state observed in the specimens. On top of that, possible room temperature grain rotation could have been taken place as observed by Rahnama et al. [39]. In order to investigate grain orientation, further electron backscattered diffraction (EBSD) analysis have to be performed and kernel average misorientation (KAM) measurements within single grains must be acquired.

## 5. Conclusions

The influence of electropulsing treatment has been investigated on prestrained UNS S32750 duplex stainless steel. It has been found that electropulsing treatments conducted on 5% and 15% prestrained specimens almost eliminate the work-hardening state in the first case, while partially recover the work-hardened state in the second. Interesting to note is the increase in yield stress and ultimate tensile strength in the case of electropulsed specimens prestrained to 15% coupled with the increase both in uniform and fracture strain.

Residual stresses did not show any particular trend: average transverse residual stress remained almost constant even though variations within the single phase was observed. Longitudinal compressive stresses values increased in the case of the specimens prestrained at 5% and electropulsed, while in the case of the 15% prestrained samples the highest compressive state was found for the material electropulsed with 500 pulses at 100 A/mm^2^.

The effect of electropulsing treatment was observed in terms of peak’s FWHM reduction of X-ray patterns. An effect in terms of recovering the work-hardened state thanks to uneven distribution and increased atomic flux due to the electrical current was hypothesized, based on the data acquired and the literature.

These results are promising in terms of replacing furnace annealing treatments with electropulsing treatments.

## Figures and Tables

**Table 1:** Chemical composition of the investigated steel (wt%).

Steel Grade	C	Si	Mn	Cr	Ni	Mo	Cu	W	P	S	N
UNS S32750	0.018	0.26	0.84	25.08	6.88	3.82	0.17	-	0.019	0.0010	0.294
